# Effects of Dietary Energy on Growth Performance, Rumen Fermentation and Bacterial Community, and Meat Quality of Holstein-Friesians Bulls Slaughtered at Different Ages

**DOI:** 10.3390/ani9121123

**Published:** 2019-12-11

**Authors:** Haibo Wang, Hang Li, Fei Wu, Xinjun Qiu, Zhantao Yu, Wenjing Niu, Yang He, Huawei Su, Binghai Cao

**Affiliations:** State Key Laboratory of Animal Nutrition, College of Animal Science and Technology, China Agricultural University, Beijing 100193, China; harper.wang@cau.edu.cn (H.W.); lihang0315@126.com (H.L.); f_wu1995@163.com (F.W.); qiuxinjun@cau.edu.cn (X.Q.); zyu18@vols.utk.edu (Z.Y.); niuwenjing4466@126.com (W.N.); he.yang@cau.edu.cn (Y.H.)

**Keywords:** dietary energy, Holstein-Friesians bull, meat quality, rumen fermentation, rumen bacterial community

## Abstract

**Simple Summary:**

Beef tenderness, flavour and juiciness are quality characteristics of consumer eating satisfaction. Marbling level of beef could determine quality characteristics by concentrating water soluble flavour molecules and releasing low fat-solubility volatiles faster. Increasing number of Holstein-Friesians bulls are used for beef production, and this breed is considered to have lower meat quality than purebred and crossbred beef cattle. Therefore, in this study we designed different dietary energy levels and slaughter ages to find ways to improve the meat quality of Holstein-Friesians bulls in beef production. We found that Holstein-Friesians bulls fed with 10.90 and 11.68 MJ/kg metabolizable energy diets, compared to a diet with 10.12 MJ/kg of metabolizable energy, had higher average daily gain, dressing percentage, intramuscular fat content and water holding capacity at 23 or 26 months of age. This study provides a basis to use bulls to produce beef by providing indicators of growth performance, rumen fermentation and ruminal bacteria community, and meat quality.

**Abstract:**

The objective of this study was to evaluate the effects of dietary energy levels on growth performance, rumen fermentation and bacterial community, and meat quality of Holstein-Friesians bulls slaughtered at different ages. Thirty-six Holstein-Friesians bulls (17 months of age) were divided into a 3 × 3 factorial experiment with three energy levels (LE, ME and HE; metabolizable energy is 10.12, 10.90 and 11.68 MJ/kg, respectively) of diets, and three slaughter ages (20, 23 and 26 months). Results indicated that bulls fed with ME and HE diets had higher dry matter intake, average daily gain, and dressing percentage at 23 or 26 months of age. The ME and HE diets also reduced bacterial diversity, altered relative abundances of bacteria and produced lower concentrations of acetate, but higher butyrate and valerate concentrations in rumen fluid. Increasing in dietary energy and slaughter age increased the intramuscular fat (IMF) and water holding capacity. In summary, Holstein-Friesians bulls fed with ME and HE diets, slaughtered at 23 and 26 months of age could be a good choice to produce beef with high IMF. Slaughter age may have less influence than dietary energy in altering fermentation by increasing amylolytic bacteria and decreasing cellulolytic bacteria, and thus, further affecting meat quality.

## 1. Introduction 

Beef tenderness, flavour and juiciness are quality characteristics of consumer eating satisfaction, of these, tenderness is the most important factor to affect the beef palatability [1,2]. Intramuscular fat (IMF) or marbling level is a characteristic appreciated by the consumer because of its positive effects on taste, tenderness and juiciness [3]. Marbling level of beef could determine quality characteristics by concentrating water soluble flavour molecules and releasing low fat-solubility volatiles faster [4,5]. However, beef from male bulls is inherently leaner than those of females or castrated with lower IMF content, which may lessen consumer liking [6]. The factors that affect the IMF deposition include breed, sex, slaughter weight and age, and nutrition [7]. 

Dietary factors, such as crude protein (CP) and carbohydrate, could affect rumen fermentation, digestibility, metabolism and meat quality [8,9]. Grain-based systems could raise growth rates and levels of carcass and muscle fat [10]. There is an increasing recognition that dairy cattle can produce excellent beef [11,12]. Holstein-Friesians is a kind of large cattle, generally considered to have a low dressing percentage [11,13]. Therefore, there is a potential to increase the meat value of Holstein-Friesians bulls by increasing the killing out percentage that are suitable for the high-value markets.

Holstein-Friesians are well suited to long-term fattening, which does not lead to excessive fat deposition or carcass quality deterioration, whereas providing insufficient feed for Holstein-Friesians extends the fattening period and decreases the level of marbling [11]. Therefore, coordinating the dietary energy and increasing the slaughter age could be good ways to use Holstein-Friesians bulls to produce highly marbled beef. Hence, we designed different dietary energy levels and slaughter ages to investigate suitable dietary energy and fattening time to make full use of Holstein-Friesians bulls for producing beef with high IMF. 

## 2. Materials and Methods

### 2.1. Animal and Experimental Design

The animal experiment was performed according to the Regulations for the Administration of Affairs Concerning Experimental Animals (The State Science and Technology Commission of China, 1988). The protocols were approved by the Laboratory Animal Welfare and Animal Experimental Ethical Committee of China Agricultural University (Permit No. AW21109102-1). The feeding trial was carried out at the Hondo Beef farm (Zhumadian, Henan Province, China). Thirty-six Holstein-Friesians bulls at an initial age of 17 months (493.3 ± 39.7 kg, body weight) were selected and randomly divided into three diet treatments according to body weight, with each treatment containing 12 replicates. The energy of the three diets were 10.12 (low energy, LE), 10.90 (medium energy, ME) and 11.68 (high energy, HE) MJ/kg, respectively. Diets were formulated to meet the nutritional requirements of beef cattle [14]. The ingredients and nutrient compositions of the experimental diets are listed in Table 1. Bulls were individually fed the total mixed rations twice daily at 7:00 and 16:30, respectively. Feed provided to each bull was recorded daily to estimate daily dry matter intake (DMI; kg/d). Bulls had free access to fresh, clean water. After being fattened for 3, 6 or 9 months, i.e., at the age of 20, 23 or 26 months, four bulls from each dietary treatment were slaughtered.

### 2.2. Samples Collection

Bulls were weighed on three consecutive days before feeding at 20, 23 and 26 months of age. Average daily gains (ADG) were calculated. At the end of each slaughter age, approximately 250 mL of rumen fluid was collected using an oesophageal tube 2 h after morning feeding. Upon reaching the slaughter age, the bulls (three treatments of 4 animals each) were transported over a distance of about 10 km to the abattoir where they were kept in lairage for 15–20 h prior to slaughter, in individual boxes with free access to water. The animals were stunned and slaughtered according to the standard protocol of slaughterhouse, then the hot carcass weight (HCW) were weighed with an online automatic weight within an accuracy of 0.5 kg. After 48 h of carcass chilling, about 10-cm-thick longissimus dorsi (LD) samples from the left half-carcass between 6th and 7th ribs were sampled and vacuum packaged. 

### 2.3. Chemical Analysis

Feedstuffs were sampled and ground weekly, and composited monthly. Composite samples were kept frozen at −20 °C prior to analysis. The Association of Official Analytical Chemists (AOAC) methods [15] were used to detect CP, ether extract and ash of feedstuffs. The Ankom method [16] was used to quantify the neutral detergent fibre (NDF).

After removing the external fat and thick connective tissue, dry matter (DM) of LD was measured using a freeze-drier (FD-1-50, Biocool, Beijing, China) for 6 d at −50 °C. The LD were analysed for CP and ether extract according to the AOAC methods [15].

### 2.4. Rumen Fermentation Parameters

A portable pH probe (HJ-90B, Aerospace Computer Company, Beijing, China) was used to measure rumen fluid pH immediately after the rumen fluid was collected. After being filtered by four layers of sterile cheesecloth, 1 mL rumen fluid was mixed with 0.25 mL of metaphosphoric acid (25 g/100 mL). Then, the volatile fatty acids (VFAs) were measured by a GC (GC-2014 Shimadzu Corporation, Kyoto, Japan). Analysis was undertaken following our previous method [17]. Briefly, controlling the column nitrogen flow rate at 46.3 cm/s, the sampling amount was 0.4 μL, and the inlet temperature was maintained at 220 °C. The initial column temperature was set at 110 °C and held for 30 s; then, the temperature was increased at 10 °C/min to 120 °C and maintained for 4 min; finally, the temperature was increased at 10 °C/min to 150 °C. The detector temperature remained at 250 °C. The concentration of rumen fluid NH_3_-N was measured according to alkaline phenol hypochlorite method [18]. A standard curve was made to know the linear relationship between the varying concentrations of ammonium sulphate standard solution and the intensity of colour produced. Briefly, 50 μL rumen fluid was mixed with 2.5 mL phenol plus nitroprusside (5 g of phenol with 25 mg of sodium nitroprusside per 500 mL of solution); then, 2 mL alkaline hydrochlorite was added (2.5 g of sodium hydroxide, 4.2 mL of sodium hypochlorite to 500 mL of solution); finally, the solution was heated at 95 °C for 5 min. Absorbance measured at room temperature at 630 nm by a spectrophotometer (UV-1700, Shimadzu Corporation, Kyoto, Japan).

### 2.5. Bacteria DNA Extraction and 16S rRNA Pyrosequencing

The DNA of bacteria in rumen fluid was extracted by using a bacterial DNA Isolation Kit (Omega Bio-Tek, Norcross, GA, USA). A Nanodrop 2000 spectrophotometer (Technologies Inc., Wilmington, DE, USA) was used to measure the concentration of DNA. The V3-V4 hypervariable region (from forward 338F to the reverse 806R) of bacterial 16S rRNA gene was amplified with the primer polymerase chain reaction. For each bacterial sample, a 10-digit barcode sequence was added to the 5′ end of the forward and reverse primers (Allwegene Company, Beijing, China). Polymerase chain reaction (PCR) was carried out using a 25 μL reaction system: 2 μL of Primer (5 μM), 12.5 μL of 2 × Taq PCR MasterMix, 3 μL of BSA (2 ng/μL), 2 μL of template DNA and 5.5 μL of RNase-free ddH_2_O. The conditions were 95 °C for 5 min, followed by 32 cycles of 95 °C for 45 s, 55 °C for 50 s and 72 °C for 45 s, with a final extension at 72 °C for 10 min. Each sample contained three PCR products, which were purified, quantified and sequenced. Eventually, high-throughput sequencing was performed via the Illumina MiSeq platform (San Diego, CA, USA). Raw data were first screened. Sequences were removed if they were shorter than 200 bp or had a quality score under 20. Qualified reads were separated using the sample-specific barcode sequences and trimmed with Illumina Analysis Pipeline Version 2.6 (Illumina, San Diego, CA, USA). Then, the dataset was analysed using Quantitative Insights Into Microbial Ecology (QIIME) (http://qiime.org/). Based on the similarity level of 97%, sequences were clustered into operational taxonomic units (OTUs) to generate rarefaction curves and to calculate the richness and diversity indices. The Ribosomal Database Project Classifier tool was used to classify all sequences into different taxonomic groups.

The raw data were submitted to Sequence Read Archive (SRA) with accession number PRJNA531244. 

### 2.6. Meat Quality

A Testo 205 pH probe (Testo AG, Schwarzwald, Germany) was used to measure the pH of LD after chilling for 48 h at 4 °C. Packed by 8 double layer filter paper, the 2.5-cm in diameter and 1-cm in thickness meat was held under 35 kg pressure for 5 min to calculate the pressing loss. Drip loss was calculated by a 2 × 3 × 5-cm LD parallel to the muscle fibre orientation hanging up for 24 h in a 4 °C sealed environment. The LD sample was heated in an 80 °C water bath to achieve a core temperature of 70 °C; then, the cooking loss was expressed as the percentage of weight loss before heating and after cooling. Six 1.27-cm diameters columnar muscle samples parallel to the muscle fibre orientation were removed from the cooked meat for measurement of the Warner-Bratzler shear force (WBSF) using a texture analyser (TA.XT plus, SMS, Godalming, Surrey, UK). The value of WBSF was calculated at an average measurement of columnar samples from each steak in Newtons.

### 2.7. Statistical Analysis

Data were statistically analysed with the general linear model procedures of SAS (SAS Inst. Inc., Cary, NC, USA). The statistical model included the effects of dietary energy levels (LE, ME or HE), slaughter ages (20, 23 or 26 months of age), and their interactions. When there was a significant interaction between dietary energy and slaughter age, post hoc testing was conducted using Duncan’s multiple comparison tests. Data were presented as the means and pooled SEM. Significant differences were declared at *p* < 0.05.

## 3. Results

### 3.1. Growth Performance

Significant differences were observed in the body weight, DMI, ADG, and feed conversion ratio (FCR) among different dietary energy levels and ages (*p* < 0.05) (Table 2). Body weight in the three dietary treatments was similar at 20 months of age, and increased at 23 and 26 months of age with the increasing dietary energy. The DMI increased at 20 and 26 months of age with the increasing dietary energy, whereas the DMI of 23-month old bulls was lowest with ME diet. The ADG of bulls fed HE diet increased at 23 months of age, and the ADG of bulls fed all the three diets increased at 26 months of age. The FCR of bulls fed with ME and HE diets decreased at 26 months of age. There was a tendency for interaction effects between dietary energy and slaughter age on the dressing percentage (*p* = 0.099) and HCW (*p* = 0.056), and the HCW increased with the increasing dietary energy and slaughter age (*p* < 0.05). Moreover, bulls fed with LE diet at 26 months of age, and bulls fed with ME and HE diets at 23 and 26 months of age had a higher dressing percentage (*p* < 0.05).

### 3.2. Rumen Fermentation Parameters

Ruminal pH, and the concentrations of NH_3_-N, isobutyrate, butyrate, isovalerate and total VFAs were not affected by the dietary energy and slaughter age (*p* > 0.05) (Table 3). The concentration of acetate and the ratio of acetate to propionate decreased, the concentrations of propionate and valerate increased with the increasing dietary energy (*p* < 0.05). In addition, the concentration of valerate increased with the increasing slaughter age (*p* < 0.05).

### 3.3. Sequencing Depth, Richness and Diversity

**Figure 1:**
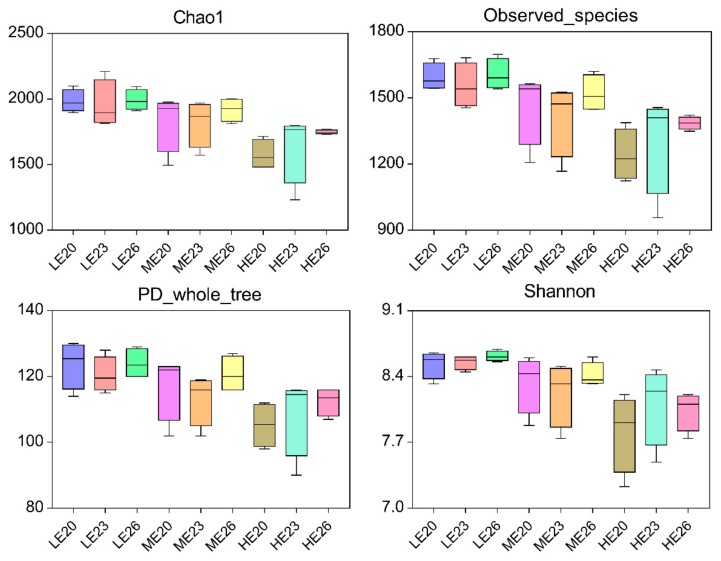
Effects of dietary energy and slaughter age on alpha diversity of the ruminal bacterial community. The top and bottom boundaries of each box represent the 75th and 25th quartile values, respectively. The horizontal lines inside each box represent median values. LE: low energy; ME: medium energy; HE: high energy. 20, 23 and 26 represent bulls slaughtered at 20, 23 and 26 months of age, respectively.

Among the 36 samples, a total of 2,232,428 valid sequences were rarefied to 62,011 sequences per sample. 27,953 reads remained after being normalised and were assigned to 1494 OTUs. The Good’s coverage indices were at least 98.4%, indicating that the rumen bacterial community was reflected accurately. Diversity indices (Chao and Observed species) and richness estimators (Phylogenetic diversity (PD) whole tree and Shannon) decreased with the increasing dietary energy (*p* < 0.05), whereas they were not influenced by the slaughter age (*p* > 0.05) (Figure 1). 

### 3.4. Taxonomic Profiles

**Figure 2:**
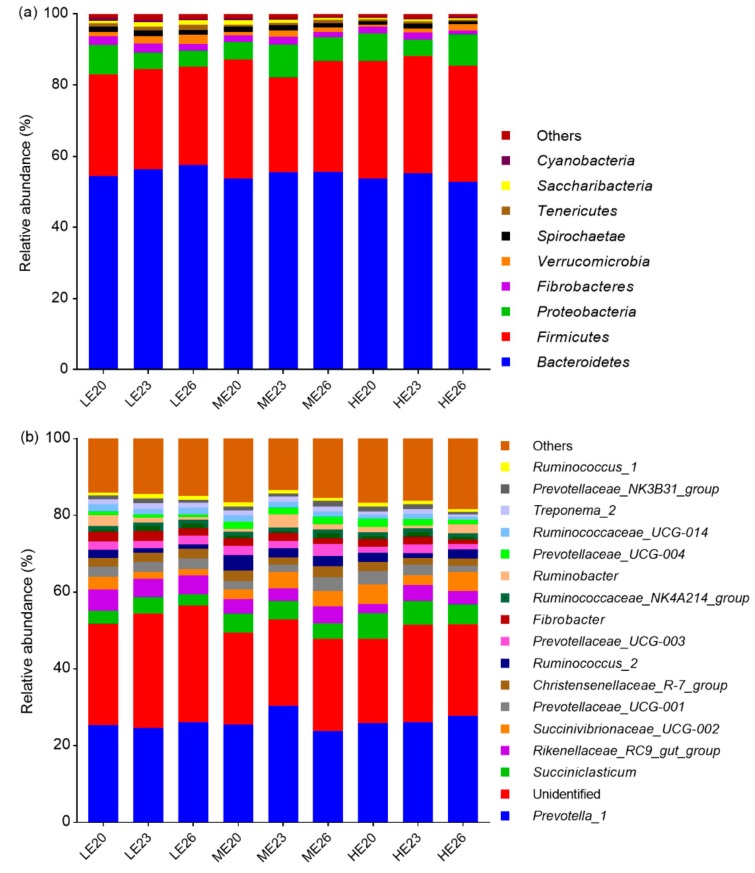
The relative abundances of ruminal bacteria at the phylum (**a**) and genus (**b**) levels. LE: low energy; ME: medium energy; HE: high energy. 20, 23 and 26 represent bulls slaughtered at 20, 23 and 26 months of age, respectively.

A total of 20 phyla were identified, the majority of which were *Bacteroidetes*, *Firmicutes*, *Proteobacteria*, *Fibrobacteres*, *Verrucomicrobia* and *Spirochaetae*, with relative abundances representing 54.62, 30.43, 6.63, 1.98, 1.45 and 1.27%, respectively (Figure 2a; Supplementary Material Table S1). The relative abundances of *Tenericutes*, *Saccharibacteria*, *Cyanobacteria* and *Lentisphaerae* decreased with the increasing dietary energy (*p* < 0.05). Slaughter age had no effects on the abundances of bacteria at the phylum level (*p* > 0.05). 

At the genus level, a total of 192 classifiable genera were detected (Supplementary Material Table S2). Relative abundances account of those were more than 1% are listed in Figure 2b. The most abundant genera in all dietary treatments were *Prevotella_1*, *Succiniclasticum* and *Rikenellaceae_RC9_gut_group*, with relative abundances that accounted for 25.73, 4.71 and 4.04%, respectively. The relative abundances of *Rikenellaceae_RC9_gut_group*, *Candidatus_Saccharimonas*, *Ruminococcaceae_UCG-010*, *Lachnospiraceae_AC2044_group* and *Lachnoclostridium_10* decreased, whereas the relative abundances of *Succiniclasticum*, *Moryella*, *Acetitomaculum*, *Eubacterium_ventriosum_group* and *Lachnoclostridium_1* increased with increasing dietary energy (*p* < 0.05). The relative abundance of *Prevotellaceae_UCG-003* was higher in bulls fed with ME diet (*p* < 0.05). In addition, the relative abundances of *Lachnospiraceae_NK3A20_group* and *Acetitomaculum* decreased, whereas the relative abundances of *Lachnospiraceae_ND3007_group* and *Ruminococcaceae_UCG-010* increased with increasing age (*p* < 0.05). There was an interaction between dietary energy and age for the relative abundance of *Oribacterium* (*p* < 0.05).

### 3.5. Meat Quality

There were no effects of dietary energy and slaughter age on the pH, cooking loss, pressing loss, drip loss and WBSF of LD (*p* > 0.05) (Table 4). There was an interaction (*p* < 0.05) between dietary energy and slaughter age observed for DM. The DM of LD in bulls fed with LE and ME diets were similar, whereas those in bulls fed with HE diet elevated at 26 months of age. The IMF contents were higher in bulls fed with ME diet at 26 months of age, and in bulls fed with HE diet at 23 and 26 months of age than others.

## 4. Discussion

Growth performance is the direct indicator to evaluate the production potential of Holstein- Friesians. In this study, the content of NDF decreased from 38.4% to 24.6%, whereas DMI increased with the increasing dietary energy. Consistent with our results, total DMI decreased sharply as NDF concentration increased over the range of 22.5% to 45.8% [19]. FCR represents the amount of feed intake divided by live weight gain, and indicates improvements in feed conversion efficiency [20]. In this study, the higher ADG and the lower FCR in bulls fed with ME and HE diets at 23 or 26 months of age, indicated that Holstein-Friesians bulls can have gain weight efficiently in the fattening period. The Holstein-Friesians bulls weighed between 600 and 650 kg, with the dressing percentages less than 55% [11,13]. The dressing percentage of bulls fed with LE diet at 26 months of age, and those of bulls fed with ME and HE bulls at 23 and 26 months of age exceed 56%, indicated that, under this conditions, Holstein-Friesians bulls had dressing percentages similar to the Angus [21].

Organic acids are the major source of energy for cattle and provide an important link between cattle and the ruminal meta-taxome. Roughages could stimulate chewing activity, which further stimulates saliva secretion. The balance between the production of fermentation acids acid and the flow of salivary buffer into rumen is a major determinant of ruminal pH [22]. In this study, both dietary energy and slaughter age had no effects on the rumen fluid pH ranging from 6.64 to 6.90, which is appropriate for growth and fermentation of rumen microorganisms [23]. Of note, 70–80% of energy requirements of ruminants come from VFA, which originate from carbohydrates via hydrolysis by ruminal microbes [24]. In this study, the concentration of propionate increased, whereas the concentration of acetate and the ratio of acetate to propionate decreased with the increasing dietary energy. This may due to that the NDF contents of LE, ME and HE diets were 38.4, 31.7 and 24.6%, respectively; hence, the decreased structural carbohydrate contributed to a decrease in acetate and increase in propionate [25]. Increases in valerate with increased dietary energy suggest that the view that diets rich in readily degradable carbohydrates is associated with changes in the propionate and valerate anabolic pathways [26]. 

The correlations of the decreased richness estimates and diversity indices with increasing dietary energy support suggestions that more efficient microbiome have lower diversity but are more specialised than less-efficient microbiome [27]. Microbial system in rumen is dominated by a core community whose structure exists regardless of effects of dietary ingredients [28]. In this study, the core community is composed of *Bacteroidetes* and *Firmicutes*. The ratio of *Firmicutes* to *Bacteroidete* is positively associated with milk yield [29]. Our results show that the ratio of *Firmicutes* to *Bacteroidete* was not affected by dietary energy. This may due to *Proteobacteria* being sensitive to low pH [30], hence, similar ruminal pH contributed to the similar ratio of *Firmicutes* to *Bacteroidete* among the dietary treatments. The relative abundances of *Tenericutes*, *Saccharibacteria*, *Cyanobacteria* and *Lentisphaerae* decreased with increasing dietary energy; these results are consistent with the reports that those phyla are involved in cellulose breakdown and produce mainly acetate [31,32,33].

*Prevotellaceae_UCG-003* belongs to the family *Prevotellaceae*, whose function is associated with ruminal starch, protein, peptide and hemicellulose fermentation [34]. In this study, the relative abundance of *Prevotellaceae_UCG-003* was higher in bulls fed with ME diet than those fed with others. This may be because the content of fibre and the release speed of peptides in bulls fed with ME diet were beneficial for the growth and reproduction of *Prevotellaceae_UCG-003*. In this study, the relative abundances of *Succiniclasticum*, *Moryella*, *Acetitomaculum*, *Eubacterium_ventriosum_group* and *Lachnoclostridium_1* increased with the increasing dietary energy, indicating that their roles involve in contributing to carbohydrate metabolic pathways. Similar to our results, *Succiniclasticum ruminis* and *Lachnoclostridium_1* have been reported to utilise energy by decarboxylating succinate to propionate, which in turn serves as a nutrient for the ruminant [35,36]. *Fibrobacter succinogenes, Ruminococcus albus, Ruminococcus flavefaciens* and *Butyrivibrio fibrisolvens* are the most abundant genera belonging to the ruminal cellulolytic bacteria, and the number of these bacteria will be decreased when the ruminal pH is less than 6.0 [37]. In this study, dietary energy had no effect on those genera except for *Ruminococcus*, which contains *Ruminococcus albus* and *Ruminococcus flavefaciens*, has been proven to decrease with decreasing substrate fibre during adaptation to a high-grain diet [38]. In addition, we detected that the relative abundances of *Ruminococcaceae_UCG-010*, *Rikenellaceae_RC9_gut_group*, *Candidatus_Saccharimonas* and *Lachnospiraceae_AC2044_group* decreased with increasing dietary energy, indicating that these genera have the function of digesting fibre [39,40]. 

The IMF could concentrate water soluble flavour molecules and release low fat-solubility volatiles faster, and further increase the perceived intensity of the flavour [41]. In this study, increasing in the dietary energy, as well as age, increased the content of IMF. Additionally, increased dietary energy increased amylolytic bacteria such as *Tenericutes*, *Saccharibacteria*, *Cyanobacteria* and *Lentisphaerae*, and decreased cellulolytic bacteria such as *Succiniclasticum*, *Moryella*, *Acetitomaculum* and *Ruminococcus*, and further increased the concentration of propionate and decreased the concentration of acetate. This may reflect the increased propionate production for bulls fed higher energy diet, which could further affect blood glucose and provide more carbon substrate for intramuscular lipogenesis [42]. Water holding capacity is attractive for consumers, which could be improved by IMF by reduced drip loss and cooking loss [43,44]. In this study, consistent with the increased IMF, the water holding capacity increased, indicated by the numerically decreased drip loss and pressing loss with increasing dietary energy. Meat tenderness, usually assessed by WBSF, is an important attribute with regard to consumption quality [45]. Our results showed that there was no difference in WBSF among the treatments. Similarly, previous studies also reported that concentrate levels [46], as well as slaughter ages [47], has no effects on cooking loss, WBSF or juiciness. 

## 5. Conclusions

Holstein-Friesians bulls fed with ME diet and slaughtered at 26 months of age, and fed with HE diet and slaughtered at 23 or 26 months of age have a better growth performance, higher dressing percentage, IMF and water holding capacity. The results suggest that changes in the diet structure including a higher energy density may affect the rumen fermentation by altering bacteria diversities and community indicated by the increasing amylolytic bacteria and decreasing cellulolytic bacteria. In summary, Holstein-Friesians bulls fed with ME and HE diets, slaughtered at 23 and 26 months of age can produce beef with high quality in terms of high IMF. In addition, compared with slaughter age, dietary energy may alter ruminal fermentation by affecting the rumen bacterial community, and further affect meat quality.

## Figures and Tables

**Table 1:** Ingredients and nutrient compositions of the experimental diets.

Item	Diet ^1^		
	LE	ME	HE
Ingredient, %			
Corn grain	24.8	40.7	57.6
DDGS ^2^	4.00	4.50	5.00
Extruded soybean	3.04	3.25	3.50
Soybean meal	3.08	2.00	0.81
Corn silage	49.9	36.5	23.0
Wheat straw	8.10	6.80	5.40
Peanut hay	6.00	4.70	2.60
Mineral-vitamin premix ^3^	0.36	0.52	0.69
NaHCO_3_	0.36	0.52	0.69
NaCl	0.36	0.52	0.69
Nutrient level			
Metabolizable energy (MJ/kg)	10.12	10.90	11.68
OM (%)	92.64	93.26	93.99
CP (%)	10.61	10.62	10.59
NDF (%)	38.39	31.71	24.59
EE (%)	3.89	4.01	4.14

^1^ LE: low energy; ME: medium energy; HE: high energy. ^2^ DDGS: distillers dried grain with solute; OM: organic matter; CP: crude protein; NDF: neutral detergent fibre; EE: ether extract. ^3^ Every kilogram of mineral-vitamin premix contained: vitamin A, 625,000 IU; Vitamin D, 100,000 IU; Fe, 6.75 g; Zn, 4.2 g; Cu, 2.5 g; Mn, 10.34 g; Co, 30 mg; I, 90 mg and Se 54 mg.

**Table 2:** Effects of dietary energy and slaughter age on growth performance of Holstein-Friesians bulls.

Item ^1^	LE			ME			HE			SEM	*p*-Value ^2^		
	20	23	26	20	23	26	20	23	26		Diet	Age	Diet × Age
DMI ^3^ (kg/d)	8.42 ^ab^	9.30 ^c^	9.97 ^d^	8.18 ^a^	8.83 ^b^	10.2 ^de^	8.72 ^b^	10.5 ^e^	12.0 ^f^	0.16	<0.001	<0.001	<0.001
ADG (kg/d)	0.95 ^a^	0.93 ^a^	1.10 ^b^	0.92 ^a^	1.00 ^ab^	1.28 ^c^	1.00 ^ab^	1.28 ^c^	1.50 ^d^	0.04	<0.001	<0.001	<0.001
FCR	8.71 ^bcd^	9.56 ^d^	9.49 ^d^	9.02 ^cd^	8.83 ^bcd^	8.07 ^ab^	8.91 ^cd^	8.33 ^abc^	7.89 ^a^	0.25	0.002	0.096	0.012
Body weight (kg)	580 ^a^	661 ^b^	799 ^d^	575 ^a^	672 ^b^	838 ^d^	583 ^a^	723 ^c^	898 ^e^	14.3	0.001	0.001	0.048
HCW (kg)	308 ^a^	354 ^b^	460 ^e^	311 ^a^	380 ^c^	484 ^e^	320 ^a^	410 ^d^	522 ^f^	14.9	<0.001	<0.001	0.056
Dressing percentage (%)	53.05 ^a^	53.50 ^ab^	57.62 ^c^	54.04 ^ab^	56.48 ^c^	57.74 ^c^	54.80 ^b^	56.69 ^c^	58.18 ^c^	0.39	0.002	<0.001	0.099

^1^ LE: low energy; ME: medium energy; HE: high energy. 20, 23 and 26 represent bulls slaughtered at 20, 23 and 26 months of age, respectively. ^2^ *p*-value: ^a–f^ Least squares means within a row lacking a common superscript differ (*p* < 0.05) due to dietary energy × slaughter age interaction. ^3^ DMI: dry matter intake; ADG: average daily gain; FCR: feed conversion ratio, the ratio of DMI to ADG; HCW: hot carcass weight.

**Table 3:** Effects of dietary energy and slaughter age on rumen fermentation parameters of Holstein-Friesians bulls.

Item ^1^	LE			ME			HE			SEM	*p*-Value		
	20	23	26	20	23	26	20	23	26		Diet	Age	Diet × Age
pH	6.73	6.74	6.89	6.67	6.71	6.83	6.85	6.70	6.69	0.08	0.687	0.378	0.231
NH_3_-N (mg/dL)	4.50	4.47	4.41	4.64	5.28	4.72	5.53	4.93	5.38	0.71	0.381	0.994	0.927
VFA ^2^ (mmol/L)													
Acetate	60.2	60.7	61.1	57.7	56.8	58.4	54.0	54.2	51.3	1.19	<0.001	0.929	0.340
Propionate	13.6	13.7	13.0	16.3	16.1	15.5	18.2	18.0	17.2	0.67	<0.001	0.294	0.999
Isobutyrate	0.61	0.59	0.59	0.58	0.60	0.57	0.65	0.61	0.60	0.04	0.475	0.698	0.966
Butyrate	7.15	6.91	7.37	6.82	7.22	7.47	8.32	7.19	7.91	0.49	0.194	0.485	0.635
Isovalerate	1.62	1.39	1.56	1.45	1.76	1.53	1.55	1.64	1.77	0.15	0.579	0.793	0.412
Valerate	0.63	0.67	0.84	0.68	0.71	0.81	0.80	0.87	0.86	0.06	0.019	0.031	0.637
Total VFA	83.8	83.9	84.5	83.5	83.2	84.3	83.5	82.6	79.7	1.33	0.133	0.767	0.373
A/P	4.42	4.44	4.73	3.56	3.57	3.79	2.98	3.02	3.00	0.18	<0.001	0.398	0.899

^1^ LE: low energy; ME: medium energy; HE: high energy. 20, 23 and 26 represent bulls slaughtered at 20, 23 and 26 months of age, respectively. ^2^ VFA: volatile fatty acid; A/P: the ratio of acetate to propionate.

**Table 4:** Effects of dietary energy and slaughter age on meat quality in longissimus dorsi of Holstein-Friesians bulls.

Item ^1^	LE			ME			HE			SEM	*p*-Value ^2^		
	20	23	26	20	23	26	20	23	26		Diet	Age	Diet × Age
pH	5.59	5.69	5.55	5.65	5.56	5.61	5.66	5.65	5.71	0.06	0.430	0.987	0.478
DM ^3^ (%)	26.6 ^ab^	26.1 ^a^	26.5 ^ab^	26.7 ^ab^	27.1 ^ab^	28.0 ^b^	26.7 ^ab^	27.0 ^ab^	31.1 ^c^	0.45	0.001	<0.001	0.002
Cooking loss (%)	31.2	31.5	31.3	29.5	29.3	29.4	30.1	30.5	28.7	2.03	0.533	0.936	0.993
Pressing loss (%)	66.6	66.7	66.3	66.2	67.9	68.7	67.9	68.5	68.7	1.65	0.460	0.772	0.962
Drip loss (%)	5.87	5.43	5.22	5.74	5.23	4.99	5.60	4.89	4.61	0.53	0.562	0.208	0.998
WBSF (N)	57.5	50.8	49.9	59.5	56.2	49.8	61.5	52.3	55.1	4.45	0.657	0.124	0.909
IMF (%)	4.43 ^a^	5.13 ^ab^	5.44 ^ab^	4.54 ^a^	5.55 ^ab^	7.17 ^c^	4.87 ^ab^	6.32 ^bc^	9.24 ^d^	0.51	0.001	<0.001	0.052
CP (%)	18.9	19.3	19.5	19.1	19.5	18.7	18.7	19.0	19.5	0.28	0.793	0.300	0.333

^1^ LE: low energy; ME: medium energy; HE: high energy. 20, 23 and 26 represent bulls slaughtered at 20, 23 and 26 months of age, respectively. ^2^ *p*-value: ^a–d^ Least squares means within a row lacking a common superscript differ (*p* < 0.05) due to dietary energy × slaughter age interaction. ^3^ DM: dry mater; WBSF: Warner-Bratzler shear force; IMF: intramuscular fat; CP: crude protein.

## Supplementary Materials

The following are available online at https://www.mdpi.com/2076-2615/9/12/1123/s1, Table S1: Relative abundance of ruminal bacteria at the phylum level; Table S2: Relative abundance of ruminal bacteria at the genus level.
