# Supplementary material for: Effects of Dietary Energy on Growth Performance, Rumen Fermentation and Bacterial Community, and Meat Quality of Holstein-Friesians Bulls Slaughtered at Different Ages
Source: Animals (Basel). 2019 Dec 11;9(12):1123. doi: 10.3390/ani9121123 (PMC6941049; doi:10.3390/ani9121123)
Supplement: Supplementary file 1 [file animals-09-01123-s001.pdf]

Effects of dietary energy on growth performance, rumen fermentation and bacterial community, and meat quality of Holstein-Friesians bulls slaughtered at different ages

Table S1. Relative abundance of ruminal bacteria at the phylum level.

Item[1]	LE			ME			HE			SEM	*P*-value		
	20	23	26	20	23	26	20	23	26		Diet	Age	Diet×Age
p__Bacteroidetes	54.1	55.9	57.1	53.3	55.2	55.3	53.4	54.8	52.4	0.812	0.623	0.718	0.946
p__Firmicutes	28.5	28.2	27.7	33.5	26.5	31.1	32.9	33.0	32.5	1.022	0.210	0.648	0.792
Firmicutes: Bacteroidetes	0.54	0.53	0.49	0.64	0.48	0.56	0.62	0.61	0.65	0.027	0.321	0.646	0.867
p__Proteobacteria	8.33	4.72	4.48	4.78	9.32	6.71	7.84	4.63	8.90	0.673	0.706	0.897	0.166
p__Fibrobacteres	2.47	2.57	1.92	1.92	2.20	1.53	1.98	2.05	1.15	0.177	0.412	0.242	0.996
p__Verrucomicrobia	1.24	2.03	2.48	1.03	1.76	1.16	0.51	1.07	1.74	0.167	0.105	0.071	0.610
p__Spirochaetae	1.42	1.54	1.41	1.39	1.46	1.24	0.84	1.33	0.80	0.091	0.104	0.397	0.918
p__Tenericutes	0.95	1.17	1.50	0.63	0.67	0.89	0.62	0.65	0.43	0.064	0.001	0.143	0.069
p__Saccharibacteria	0.77	1.20	1.19	1.23	0.86	0.58	0.35	0.54	0.35	0.084	0.003	0.640	0.153
p__Cyanobacteria	0.77	0.62	0.52	0.57	0.39	0.26	0.26	0.31	0.45	0.047	0.023	0.489	0.347
p__SR1_Absconditabacteria	0.28	0.81	0.53	0.47	0.19	0.24	0.10	0.63	0.14	0.060	0.098	0.094	0.074
p__Synergistetes	0.33	0.31	0.16	0.25	0.37	0.38	0.25	0.30	0.22	0.026	0.443	0.591	0.478
p__Actinobacteria	0.21	0.17	0.17	0.37	0.49	0.21	0.61	0.12	0.21	0.045	0.236	0.150	0.159
p__Lentisphaerae	0.34	0.39	0.52	0.18	0.32	0.18	0.07	0.21	0.33	0.038	0.038	0.223	0.600
p__Elusimicrobia	0.22	0.35	0.27	0.25	0.15	0.15	0.15	0.31	0.21	0.030	0.441	0.639	0.704

[1]LE: low energy; ME: medium energy; HE: high energy. 20, 23 and 26 represent bulls slaughtered at 20, 23 and 26 months of age, respectively.

Table S2. Relative abundance of ruminal bacteria at the genus level.

Item[1]	LE			ME			HE			SEM	*P*-value		
	20	23	26	20	23	26	20	23	26		Diet	Age	Diet×Age
g__*Prevotella_1*	24.9	24.1	25.6	25.1	30.0	23.4	25.5	25.8	27.3	0.999	0.860	0.838	0.702
g__Unidentified	26.5	29.9	30.4	24.0	22.4	24.0	22.0	25.3	24.0	0.764	0.004	0.438	0.679
g__*Succiniclasticum*	3.38	4.28	2.98	4.89	4.85	4.13	6.66	6.09	5.17	0.349	0.022	0.428	0.958
g__*Rikenellaceae_RC9_gut_group*	5.49	4.69	4.92	3.79	3.17	4.32	2.32	4.17	3.46	0.210	0.001	0.659	0.054
g__*Succinivibrionaceae_UCG-002*	3.35	1.82	1.63	2.51	4.35	4.05	5.09	2.58	4.88	0.462	0.248	0.796	0.513
g__*Prevotellaceae_UCG-001*	2.67	2.57	2.81	2.20	1.90	3.63	3.46	2.70	1.67	0.199	0.975	0.688	0.083
g__*Christensenellaceae_R-7_group*	2.24	2.45	2.44	2.70	1.89	2.85	2.50	1.86	2.03	0.136	0.586	0.424	0.625
g__*Ruminococcus_2*	2.10	1.18	1.15	4.02	2.38	2.63	2.32	1.29	2.28	0.271	0.067	0.184	0.913
g__*Prevotellaceae_UCG-003*	2.15	1.89	2.23	2.49	1.92	3.22	1.60	2.18	1.40	0.126	0.016	0.533	0.054
g__*Fibrobacter*	2.47	2.56	1.91	1.92	2.19	1.52	1.98	2.05	1.14	0.177	0.417	0.239	0.996
g__*Ruminococcaceae_NK4A214_group*	1.56	2.24	2.27	1.68	1.28	2.19	1.70	2.09	1.63	0.127	0.624	0.486	0.395
g__*Ruminobacter*	2.88	1.26	0.74	0.77	3.42	1.35	1.43	0.76	2.36	0.297	0.895	0.896	0.063
g__*Prevotellaceae_UCG-004*	0.92	0.84	0.74	1.88	1.86	1.97	2.22	1.65	1.22	0.182	0.052	0.711	0.894
g__*Ruminococcaceae_UCG-014*	1.84	1.44	1.66	1.63	1.39	1.45	0.99	1.48	0.74	0.121	0.158	0.787	0.665
g__*Treponema_2*	1.33	1.45	1.32	1.31	1.39	1.18	0.79	1.25	0.71	0.088	0.104	0.382	0.922
g__*Prevotellaceae_NK3B31_group*	0.96	1.32	0.78	0.94	0.81	1.48	1.24	1.15	0.49	0.103	0.895	0.773	0.151
g__*Ruminococcus_1*	0.84	1.11	0.96	1.18	0.87	0.87	1.10	0.91	0.80	0.058	0.959	0.546	0.540
g__*Eubacterium_coprostanoligenes_group*	0.71	0.70	0.83	0.95	0.76	0.83	0.80	1.27	1.00	0.050	0.067	0.737	0.199
g__*Candidatus_Saccharimonas*	0.76	1.20	1.19	1.23	0.86	0.58	0.35	0.54	0.35	0.084	0.003	0.640	0.156
g__*Phocaeicola*	0.86	0.67	0.62	0.68	0.66	0.96	0.88	0.80	0.43	0.047	0.827	0.449	0.067
g__*Lachnospiraceae_NK3A20_group*	0.62	0.47	0.51	1.09	0.60	0.52	1.22	0.70	0.63	0.062	0.058	0.004	0.517
g__*Lachnospiraceae_ND3007_group*	0.49	0.79	1.01	0.29	0.43	0.94	0.97	0.49	0.93	0.063	0.130	0.006	0.076
g__*Saccharofermentans*	0.70	0.61	0.83	0.92	0.65	0.71	0.67	0.42	0.70	0.039	0.201	0.056	0.585
g__*Moryella*	0.46	0.47	0.49	0.68	0.54	0.73	0.96	0.66	0.65	0.044	0.029	0.365	0.522
g__*Ruminococcaceae_UCG-001*	0.10	0.10	0.02	0.10	0.14	0.04	0.29	0.15	4.42	0.480	0.336	0.431	0.435

g__Ruminococcaceae_UCG-005	0.45	0.60	0.55	0.60	0.52	0.53	0.60	1.04	0.45	0.054	0.409	0.245	0.303
g__Ruminococcaceae_UCG-010	0.53	0.76	0.86	0.50	0.55	0.75	0.26	0.54	0.57	0.042	0.017	0.006	0.830
g__Papillibacter	0.53	0.54	0.68	0.44	0.57	0.88	0.43	0.47	0.46	0.041	0.166	0.097	0.460
g__Veillonellaceae_UCG-001	0.42	0.32	0.39	0.40	0.53	0.48	0.49	0.56	0.52	0.031	0.178	0.893	0.792
g__Erysipelotrichaceae_UCG-004	0.31	0.30	0.44	0.20	0.33	0.35	0.40	0.99	0.50	0.089	0.305	0.582	0.761
g__Eubacterium_ruminantium_group	0.30	0.32	0.32	0.60	0.33	0.46	0.59	0.37	0.35	0.036	0.190	0.175	0.573
g__Butyrivibrio_2	0.36	0.39	0.39	0.28	0.31	0.43	0.46	0.40	0.32	0.025	0.726	0.967	0.491
g__Pseudobutyrivibrio	0.32	0.42	0.49	0.32	0.32	0.32	0.40	0.38	0.31	0.032	0.565	0.937	0.782
g__Lachnospiraceae_XPB1014_group	0.32	0.27	0.35	0.47	0.40	0.34	0.33	0.23	0.37	0.028	0.348	0.560	0.774
g__Lachnospiraceae_AC2044_group	0.37	0.38	0.35	0.37	0.24	0.21	0.19	0.18	0.20	0.021	0.001	0.422	0.416
g__Anaeroplasma	0.25	0.35	0.36	0.20	0.23	0.34	0.22	0.27	0.19	0.018	0.090	0.157	0.313
g__Pyramidobacter	0.30	0.29	0.14	0.21	0.35	0.36	0.23	0.26	0.19	0.026	0.451	0.536	0.438
g__Anaerovorax	0.22	0.23	0.24	0.28	0.20	0.34	0.29	0.25	0.20	0.015	0.428	0.495	0.206
g__Acetitomaculum	0.19	0.17	0.14	0.38	0.21	0.17	0.45	0.19	0.30	0.023	0.008	0.003	0.193
g__Ruminococcaceae_UCG-002	0.10	0.15	0.05	0.26	0.14	0.04	0.10	0.68	0.52	0.065	0.065	0.520	0.316
g__Anaerotruncus	0.14	0.21	0.26	0.22	0.17	0.32	0.22	0.21	0.19	0.015	0.560	0.148	0.199
g__Elusimicrobium	0.19	0.31	0.21	0.20	0.11	0.09	0.10	0.28	0.15	0.028	0.358	0.450	0.627
g__Eubacterium_ventriosum_group	0.12	0.09	0.10	0.29	0.09	0.39	0.26	0.11	0.05	0.029	0.046	0.130	0.109
g__Ruminococcaceae_V9D2013_group	0.17	0.22	0.16	0.21	0.03	0.22	0.04	0.26	0.03	0.028	0.585	0.834	0.103
g__Ruminiclostridium_9	0.12	0.23	0.08	0.29	0.05	0.19	0.08	0.17	0.14	0.039	0.893	0.970	0.590
g__Succinimonas	0.26	0.18	0.09	0.12	0.24	0.13	0.08	0.07	0.16	0.021	0.308	0.757	0.188
g__Uncultured	0.24	0.05	0.05	0.56	0.04	0.07	0.05	0.12	0.13	0.053	0.565	0.181	0.336
g__Probable_genus_10	0.22	0.16	0.13	0.09	0.13	0.10	0.13	0.23	0.08	0.021	0.478	0.404	0.722
g__Desulfovibrio	0.15	0.10	0.16	0.15	0.11	0.17	0.18	0.10	0.15	0.009	0.934	0.018	0.909
g__Bifidobacterium	0.05	0.04	0.09	0.15	0.22	0.11	0.40	0.02	0.15	0.033	0.214	0.333	0.147
g__Ruminococcaceae_UCG-013	0.07	0.09	0.11	0.17	0.15	0.08	0.09	0.25	0.19	0.022	0.291	0.642	0.573
g__Marvinbryantia	0.12	0.10	0.08	0.14	0.05	0.24	0.12	0.19	0.15	0.022	0.609	0.741	0.469
g__Clostridium_sensu_stricto_1	0.13	0.08	0.04	0.05	0.08	0.02	0.08	0.36	0.17	0.040	0.283	0.598	0.723
g__Selenomonas_1	0.07	0.03	0.04	0.06	0.10	0.05	0.33	0.13	0.20	0.031	0.045	0.616	0.762

g__Lachnoclostridium_10	0.23	0.17	0.18	0.08	0.09	0.11	0.03	0.05	0.03	0.016	0.000	0.942	0.791
g__Prevotellaceae_YAB2003_group	0.14	0.11	0.10	0.06	0.13	0.07	0.18	0.12	0.06	0.018	0.684	0.533	0.724
g__Oribacterium	0.10	0.07	0.10	0.10	0.09	0.14	0.17	0.12	0.08	0.008	0.134	0.166	0.031
g__Lachnoclostridium_1	0.03	0.03	0.04	0.08	0.08	0.08	0.33	0.15	0.10	0.020	0.000	0.115	0.049

[1]LE: low energy; ME: medium energy; HE: high energy. 20, 23 and 26 represent bulls slaughtered at 20, 23 and 26 months of age, respectively.
